# Environmental factors shaping cancer outcomes in Alabama: A scoping review

**DOI:** 10.1007/s11764-026-02061-x

**Published:** 2026-07-11

**Authors:** Anita Aboagye, Nicole Caviness-Ashe, Miriam A. Miles, Oluseun Akinyele, Abigail A. Ninson, Cha’Breia Means, Lindsay Aaron-Wade, Mary A. Aboagye, Emma O’Hagan, Elizabeth Baker, Mackenzie E. Fowler, Stephen Sodeke, Elizabeth E. Brown, Kathryn A. Kaiser, Timiya S. Nolan

**Affiliations:** 1 Heersink School of Medicine, University of Alabama at Birmingham, ALGEN 687, 701 19 St South, Birmingham, AL 35294, USA; 2 O’Neal Comprehensive Cancer Center, University of Alabama at Birmingham, Birmingham, AL, USA; 3 The Embrace Society, East Legon, Accra, Ghana; 4 School of Nursing, University of Alabama at Birmingham, Birmingham, AL, USA; 5 School of Health Professions, University of Alabama at Birmingham, Birmingham, AL, USA; 6 Lister Hill Library of Health Science, University of Alabama at Birmingham, Birmingham, AL, USA; 7 School of Public Health, University of Alabama at Birmingham, Birmingham, AL, USA; 8 Center for Biomedical Research, Tuskegee University, Tuskegee, AL, USA

**Keywords:** Cancer survivors, Alabama, Environmental exposure, Industrial development, Risk assessment, Economic factors

## Abstract

**Purpose:**

This scoping review explores how environmental, social, and structural factors influence cancer outcomes in Alabama. Using the Robison and Hudson (2014) survivorship framework, we examined how individual, healthcare provider, and system-level factors influence cancer vulnerability across communities in Alabama.

**Methods:**

Following PRISMA-ScR reporting and Joanna Briggs Institute guidelines, we identified and reviewed peer-reviewed studies published between 1995 and 2024 across five databases (PubMed, Embase, Scopus, CINAHL, and PsychINFO). Twenty-one studies met the inclusion criteria and were analyzed across six key areas: food and neighborhood environment, exposure assessment and environmental measurement methods, industrial/toxic releases as exposures, legacy/historical exposures and hematologic outcomes, socioeconomic environment and cancer incidence and racial disparities, community burden, and environmental risk.

**Results:**

Cancer disparities in Alabama are closely tied to geographic, racial, and economic factors. Communities in the Black Belt and industrial corridors face higher exposure to environmental hazards due to historical segregation and industrial development patterns. These exposures are linked to increased rates of colorectal, ovarian, and blood cancers, particularly in areas with persistent poverty and limited access to healthcare.

**Conclusions:**

Cancer outcomes in Alabama cannot be separated from the broader social and environmental context. Understanding these connections is essential for addressing the drivers’ underlying health disparities.

**Implications for Cancer Survivors:**

Improving cancer survivorship in Alabama requires a comprehensive approach that includes environmental risk assessments, enhanced provider education, and community-driven prevention. These approaches may include local screening initiatives, environmental cleanup, and targeted outreach to high-risk populations.

Cancer remains one of the leading causes of mortality worldwide, with nearly 20 million new cases and 9.7 million deaths reported in 2022 [1]. By 2050, the total number of cancer cases is expected to reach 35.3 million, representing a 76.6% increase from the 2022 estimate of 20 million. In the same period, cancer-related deaths are projected to rise to 18.5 million, an 89.7% increase from the 2022 estimate of 9.7 million [2]. In the USA, cancer continues to pose a major public health challenge [3]. Alabama ranks 46th out of 50 states in overall health, with cancer being the second leading cause of death. Moreover, compared to national averages, Alabama faces persistent socioeconomic and demographic vulnerabilities. Alabama’s population is older (18.2% are 65 years or older vs. 17.7% in the USA), less educated (10.8% with less than high school education vs. 10.3% in the USA), has lower household income (median household income $62,212 vs. $77,719 in the USA) and higher poverty (15.6% in poverty vs. 12.5% in the USA) [3], contributing to its elevated cancer burden and cancer disparities [4].

Cancer outcomes, including incidence, stage at diagnosis, survival, quality of health, and overall health, remain influenced by a range of clinical, behavioral, and environmental factors [5–8]. Environmental exposures are recognized as important and modifiable determinants of cancer outcomes [9]. The National Institute of Environmental Health Sciences (NIEHS) defines the environment as encompassing multiple interrelated domains that influence health outcomes: the physical environment (air, water, soil, and climate), the chemical environment (exposures to pollutants and pesticides), the biological environment (microorganisms and biological agents), the built environment (housing, transportation systems, and urban infrastructure), and the social environment (socioeconomic conditions, cultural influences, and social support structures) [10]. These dimensions are inter-related and influence cancer risk and outcomes, resilience, and health inequities across diverse populations [5, 9, 11].

Centuries of research have established that environmental risks such as exposure to pesticides, industrial chemicals, air pollutants, and occupational toxins significantly contribute to cancer incidence and mortality [12–14]. Early investigations by John Hill in 1761 and Sir Percival Pott in 1775 were the first to link environmental exposures to cancer [15, 16]. Building on these early findings, Doll and Peto (1981) estimated that between eighty and ninety percent of human cancers could be attributed to environmental and lifestyle factors, identifying tobacco, diet, occupational exposures, infections, and pollution as key contributors to preventable cancer risk [17]. Long-term exposure to fine particulate matter (PM2.5) has been consistently linked to increased risk of cancer. Evidence from the American Cancer Society’s Cancer Prevention Study II, which followed approximately 500,000 adults across US metropolitan areas, demonstrated that each 10 μg/m^3^ increase in PM2.5 was associated with an 8% increase in lung cancer mortality [18]. Additional analysis among lifelong never-smokers found similar associations, with a 15–27% increase in lung cancer mortality per 10 μg/m^3^ increment in PM2.5 [19]. Comparable associations have been identified for hormonally mediated cancers, with higher PM2.5 exposure linked to an increased risk of estrogen receptor-positive breast cancer [20]. In addition, data from the National Health and Nutrition Examination Survey (NHANES) show that biomarkers of per- and polyfluoroalkyl substances (PFAS), phenols, and parabens are associated with higher odds of melanoma, ovarian, and uterine cancers among women [21].

Residential proximity to hazardous waste facilities, industrial zones, and pesticide-intensive agricultural areas has also been associated with increased risk of childhood leukemia, birth defects, and other malignancies [22, 23]. Complementing these population-level findings, occupational studies of chemical manufacturing workers demonstrate significantly elevated risks for respiratory system cancers, including cancers of the lung, pleura, larynx, stomach, kidney, and non-Hodgkin lymphoma, particularly with long employment durations and latency periods over 30 years [11].

The built and social environments further modify both exposure and the capacity for prevention. Communities characterized by poverty, limited access to healthcare and screening, and weak neighborhood infrastructure experience higher cancer incidence and mortality [22]. Socioeconomic disadvantage, housing instability, and transportation barriers restrict access to early detection and treatment, resulting in delayed diagnosis and poorer outcomes. Counties with higher obesity-related cancer mortality tend to have larger proportions of non-Hispanic Black residents, older populations, higher poverty rates, and greater prevalence of obesity and diabetes. Limited access to healthy foods, reflected in higher food desert and food swamp scores, has been associated with seventy-seven percent higher odds of obesity-related cancer mortality [22].

Environmental justice research highlights that these disparities are not random but structured by social and political forces. Morello-Frosch and colleagues (2002) demonstrated that communities of color disproportionately bear the burden of industrial pollution, driven primarily by facility siting decisions rather than population movement [11]. Similarly, Brender, Maantay, and Chakraborty (2011) synthesized evidence from 94 studies showing that individuals living near hazardous sites face increased risks of cancer, with racial and socioeconomic inequalities amplifying vulnerability [23]. Krieger’s ecosocial framework and Bullard’s foundational work on environmental racism highlight that social power and policy decisions determine both the distribution of exposure and the ability of communities to respond to risk (20).

Building on this evidence, the implications of environmental inequities are particularly pronounced in the southern USA, where industrial legacy, poverty, and racial segregation converge to shape cancer risk. Alabama provides a critical setting for examining these interconnections (see Fig. 1 for Alabama cancer incidence rates).

Despite extensive national research linking environmental and social determinants to cancer outcomes, few studies have synthesized evidence specific to Alabama. This scoping review addresses that gap by examining existing literature on environmental risks and cancer outcomes among Alabamians.

## Framework

This review was guided by the Robison and Hudson (2014) framework [24], which characterizes cancer outcomes through the interaction of patient, disease, provider, and health system factors. The framework was adapted to align with our research question and the research objectives (Fig. 2). The framework guided our data extraction and interpretation of how environmental exposures, social challenges, and healthcare access affect cancer risk and survival in the state of Alabama. The framework is also guided by the mapping of themes such as food and neighborhood environment, exposure assessment and environmental measurement methods, industrial/toxic releases as exposures, and socioeconomic environment, showing how these factors influence both disease and long-term survivorship (Fig. 2).

## Methods

### Literature search

This scoping review followed the PRISMA-ScR reporting checklist and Joanna Briggs Institute (JBI) methodology. The protocol was registered with Open Science Framework in November 2025 [25]. A trained information specialist supported the search strategy. We searched Medline (PubMed), Embase (Elsevier), Scopus (Elsevier), Cumulative Index of Nursing and Allied Health Literature Plus with Full Text (EBSCOhost), and APA PsychINFO (EBSCO-host) databases to identify peer-reviewed studies published between January 1, 1995, and December 30, 2024. Eligible studies focused on environmental, social, or structural factors influencing cancer outcomes in Alabama. The full literature search was conducted on January 2, 2025. We excluded abstracts, opinion papers, or grey literature and studies that were not in English.

### Study selection

A total of 3556 articles were screened using Covidence. After removing duplicates, 1757 titles and abstracts were reviewed independently by two team members. Full-text review was conducted for 328 articles, with 216 meeting the inclusion criteria. Disagreements were resolved by consensus. A PRISMA flow diagram summarizes the process (Fig. 3).

### Inclusion and eligibility criteria

Included studies met the following criteria: (1) full-text peer-reviewed, (2) delineated results and findings for residents of Alabama, and (3) addressed cancer health outcomes applicable to the life experiences of patients or participants (i.e., individual, population, clinical, and/or health-system level outcomes). We excluded studies that (1) were considered grey literature, including dissertations, commentaries, editorials, conference proceedings, and abstracts (2) did not report findings applicable to patient or participant life experiences (i.e., laboratory, animal, or cellular studies, etc.), and (3) studies that utilized the same dataset and reported no new information relative to cancer health outcomes for residents of Alabama.

### Data extraction and appraisal

Data extraction proceeded in four structured stages. First, all studies were categorized according to an adapted Robison and Hudson framework: cancer burden, environmental risk factors, cancer prevention, and access to care. Five to six exemplar extractions were completed collaboratively to ensure consistency. Extracted data included publication year, study purpose, population characteristics, sample size, study design, relevant cancer outcomes, themes, and funding sources.

Independent reviewers (NCA, AA, CM, AN, LAW) completed data extractions across all matrices. Each extraction was cross-reviewed by two additional reviewers to ensure accuracy and transparency. The JBI Critical Appraisal Checklists were applied to assess methodological rigor; however, no studies were excluded based on appraisal scores. Final verification and consensus were reached through team discussion.

### Synthesis

Findings were synthesized narratively to identify patterns across environmental, social, and structural factors using the Robison and Hudson framework to highlight how exposures and healthcare access shape cancer outcomes in Alabama.

## Results

### Study characteristics

Twenty-one studies met the inclusion criteria for this scoping review. Study characteristics and methodological quality are summarized in Table 1. The studies span more than two decades (1995–2024) and encompass a wide range of cancer types, including colorectal, cervical, ovarian, hematologic malignancies, melanoma, and multi-cancer or statewide analyses. Most investigations employed quantitative or population-based study designs using cancer registry data, administrative databases, or geospatial modeling. Several studies incorporated qualitative or mixed methods approaches to capture community perspectives. The geographic focus included both urban and rural populations across Alabama, with particular attention to the Black Belt region, an area historically marked by economic hardship, limited healthcare access, and elevated cancer burden. Some studies compared Alabama outcomes with neighboring Deep South states or examined regionspecific effects of industrial, environmental, and socioeconomic exposures. Collectively, these studies illustrate how environmental, structural, and demographic factors converge to influence cancer risk, stage at diagnosis, treatment adherence, and survivorship outcomes. To further contextualize the breadth of cancer burden identified across studies, Table 2 summarizes predominant cancer types and commonly reported environmental, social, and structural determinants associated with cancer outcomes in Alabama.

### Research participants

Of the studies reviewed, approximately 76% (*n* = 16) focused exclusively on participants residing in Alabama. The remaining 24% (*n* = 5) included multi-state analyses that incorporated Alabama, often capturing counties within the Black Belt region. Sample sizes varied widely. Qualitative studies included between 9 and 145 participants, while population-based or registry studies ranged from 284 to 37,864 individuals. Most studies concentrated on African American, female, or rural populations. Occupational cohorts comprised 652 to 3512 industrial workers, and pediatric studies included children and adolescents diagnosed with leukemia and other cancers. Participants ranged in age from early childhood to older adulthood.

### Food and neighborhood environment

Daily living conditions across Alabama significantly influence cancer susceptibility, detection, and survivorship. In 12 Black Belt cities, field audits of 61 food retail outlets revealed a retail environment dominated by convenience stores offering inexpensive, calorie-dense foods, while supermarkets and farmers’ markets were scarce. Healthier options such as fruits, vegetables, and low-fat products were limited, and health-promotion messaging was minimal or absent [26]. This scarcity reflects broader structural constraints, where poverty and limited transportation turn diet into a form of environmental exposure. Community perspectives deepen this understanding. In Walker County, residents identified high food costs, lack of insurance, and weak primary care infrastructure as interconnected barriers to health. Cancer, obesity, and diabetes were viewed as outcomes of the same deprived environment [27]. Perceptions of risk varied within communities. In a predominantly Black neighborhood in Montgomery, women expressed greater concern about pollution and its financial impact than men, while lower education levels were associated with higher perceived risk but reduced capacity to act [28].

Access to care further compounds these challenges. Rural residents in the Deep South travel significantly farther than urban counterparts for oncology services, with prostate cancer patients facing the longest commutes [29]. These travel demands contribute to delays and incomplete treatment. Even after treatment, geography continues to influence outcomes. Patients who underwent endoscopic mucosal resection showed notable differences in follow-up adherence based on education and travel distance. Those traveling moderate distances were most likely to return for a colonoscopy, suggesting that transportation, motivation, and health literacy interact to shape compliance [30]. Additionally, pediatric studies emphasize the consequences of geographic disparity. Children living more than 100 miles from tertiary care centers were significantly more likely to die within five years of diagnosis. These counties were marked by persistent poverty and predominantly Black populations [31, 32]. For these families, long travel distances, weak infrastructure, and limited local care function as chronic exposures that affect both physiological resilience and continuity of treatment. Taken together, the evidence portrays an Alabama where food access, transportation, and healthcare distribution are tightly interwoven with cancer outcomes.

Viewed through the Robison and Hudson framework, findings illustrate how patient-level factors such as dietary behavior, stress, and motivation interact with system-level factors including service availability, distance, and affordability [24]. This constrained agency within an under-resourced system creates cumulative disadvantage, where poor nutrition increases baseline cancer risk and limited access undermines cancer diagnosis, treatment, and survivorship.

### Exposure assessment and environmental measurement methods

A second group of studies focused on refining how environmental risk factors are measured and associated with outcomes among cancer survivors. By integrating emission inventories with cancer registry data, researchers identified special geographic clustering of ovarian cancer incidence in regions with historical pulp and paper mill emissions, identifying statistically significant clusters in counties with high waterborne emissions [33]. Although the analysis focused on cancer incidence rather than post-diagnosis outcomes, these exposure assessment methods are directly relevant to survivorship by identifying populations likely to experience cumulative environmental risk, informing long-term surveillance, secondary cancer prevention, and resource allocation for survivors residing in a historically contaminated region [33].

Spatial autocorrelation techniques demonstrated that cancer and exposure are not randomly distributed, strengthening the case for biologic plausibility for associations. Analyses conducted in national datasets were used to identify multiple myeloma hot spots in the Southeast, connecting current cancer patterns to cumulative environmental burden [34]. Socioeconomic and healthcare resource metrics were incorporated into geographic population-based models examining variation in colon cancer incidence and stage at diagnosis. Counties with fewer physicians, lower educational attainment, and higher poverty, particularly among Black residents, had higher colon cancer incidence and more late-stage diagnoses, suggesting the role of healthcare access and socioeconomic context, rather than environmental exposure, in shaping disparities [35]. State cancer registries were shown to be resilient tools for estimating cancer burden even during population disruptions, such as those caused by Hurricane Katrina [36].

Spatial detection methods flagged rare cancers as well. Mapping of uveal melanoma clusters revealed localized accumulations that, although not statistically conclusive, warranted environmental investigation and community engagement [37]. These early signals demonstrate how spatial analytics can guide targeted inquiry and support public reassurance. Industrial exposure studies provided quantitative depth to these ecological insights. Cumulative dose modeling linked 1,3-butadiene exposure to leukemia risk, estimating environmental concentrations associated with specific excess risk levels [38]. Occupational cohorts of fluorochemical plant workers showed elevated rates of liver and biliary tract cancers, reinforcing evidence of systemic toxicity [39, 40]. These findings demonstrate how workplace data can inform community exposure models and connect laboratory toxicology with increased cancer risk.

Although these studies show progress in environmental assessment, challenges remain. Exposure histories are often incomplete, latency periods obscure temporal trends, and mixed pollutants complicate independent effects. Nonetheless, the integration of registry data, emissions monitoring, and geospatial analytics offers a strong foundation for risk assessment. Within the Robison and Hudson framework, these methodological advances highlight system-level factors such as surveillance infrastructure and data quality, and provider-level factors where clinicians rely on accurate data to guide risk communication and follow-up care [24].

### Industrial/toxic releases as exposures

Industry shapes much of Alabama’s environmental landscape, and its health impact is evident across multiple studies. Geographic analyses revealed that counties with dense pulp and paper operations and high waterborne emissions experienced elevated ovarian cancer incidence, aligning disease patterns with manufacturing geography [33]. Occupational cohort analyses of fluorochemical manufacturing workers in Decatur identified elevated mortality from bladder cancer among employees who held high-exposure jobs, indicating increased cancer risk associated with industrial work environments [39]. These findings were reinforced by analyses of healthcare utilization among chemical plant workers, which showed higher rates of episodes of care for malignant neoplasms, benign colon polyps, and selected urologic conditions compared with film-plant controls, particularly among long-term high-exposure employees [40]. Although these episode-of-care data do not provide definitive measures of cancer risk, they highlight morbidity patterns consistent with occupational exposure to industrial toxicants. Together, these studies demonstrate that industrial facilities function as concentrated sources of chemical exposure, placing workers and surrounding communities at heightened risk of adverse cancer-related outcomes. Occupational cohort analyses of chemical manufacturing workers identified statistically significant associations between cumulative 1,3-butadiene exposure and all leukemia combined in primary models; however, findings for specific subtypes such as chronic lymphocytic leukemia were less consistent across models [38].

Cancer exposure is not solely chemical; it is also social. Qualitative interviews in North Mobile County revealed that residents and plant workers acknowledged elevated cancer and chronic disease risk associated with nearby industrial activity, yet refrained from protest due to fear of losing stable industrial employment [41]. Economic dependency perpetuated silence, embedding environmental injustice into local culture [41]. The same factories that provide income also produce pollutants, creating conflict between health and livelihood.

These studies present Alabama’s industrial corridors as zones of cumulative risk, where toxic emissions, occupational hazards, and socioeconomic vulnerability intersect. Mitigating these risks requires both environmental remediation and socioeconomic transition to reduce reliance on hazardous industries. Within the Robinson and Hudson framework, industrial toxic exposures connect cancer-related outcomes with patient-level factors such as employment-related risk perception and economic dependence, alongside system-level variability in occupational health surveillance and regulatory oversight [24].

### Legacy/historical exposures and hematologic outcomes

Several studies demonstrate that cancer risk in Alabama reflects environmental and occupational exposures accumulated over extended periods, with effects that emerge after long latency. Using exposure-response modeling, Sielken and Valdez-Flores (2015) quantified lifetime added risk of leukemia and lymphoid neoplasms associated with cumulative 1,3-butadiene exposure, modeling both environmental exposure from birth and occupational exposure across a typical working lifetime [38]. This modeling highlights how modest but sustained exposure can translate into excess hematologic cancer risk decades later. Complementing this risk modeling, long-term follow-up of fluorochemical manufacturing workers in Decatur revealed increased mortality from bladder cancer among employees with prolonged high-exposure job histories, highlighting the delayed manifestation of exposure-related malignancy [39]. Although overall cancer mortality was not elevated, excess risk was concentrated among workers with extended tenure in high-exposure roles, consistent with latency-driven effects. Further evidence of legacy exposure was observed in analyses of healthcare utilization patterns among chemical plant workers, where elevated episodes of care for malignant neoplasms, benign colon polyps, and selected urologic conditions were more frequent among long-term high-exposure employees compared with film-plant controls [40]. Per fluorinated compounds persist in human tissue and local ecosystems, thus the health burden can continue long after emissions decline, linking present-day morbidity to industrial decisions made decades ago [42].

Spatial analyses provide converging evidence at the population level. Counties with long-standing pulp and paper industries showed excess ovarian cancer incidence, whereas multiple myeloma clusters were most pronounced in southeastern regions with a history of industrial and agricultural chemical use [33, 34]. These patterns suggest that legacy pollutants remain biologically active in soil, water, and air.

In communities where families have lived and worked for generations near these sites, environmental exposure is inherited alongside property and culture. For residents without the means to relocate, cumulative contact with contaminants functions as a life-course exposure that may overlap with treatment-related risks among cancer survivors. These findings suggest that surveillance systems should be capable of tracing exposure histories across decades and linking them to current clinical records. Within the Robison and Hudson framework, legacy exposures reflect the intersection of cancer-related factors such as long disease latency and the emergence of secondary malignancies with system-level processes, including long-term surveillance and cancer registry integration [24]. Recognition of these delayed toxic effects depends on sustained provider awareness and accurate patient histories, which together enable earlier identification of morbidity among long-term survivors.

### Socioeconomic environment and cancer incidence

Socioeconomic deprivation emerged as one of the most consistent predictors of cancer burden in Alabama. Counties with lower educational attainment, greater poverty, and fewer physicians had higher colon cancer incidence and a greater proportion of distant-stage diagnoses, particularly among Black residents [35]. Poverty also influences survival among patients with cancer. In a statewide pediatric cohort, AYA living in counties with persistent child poverty were significantly more likely to die within five years of diagnosis. These children were predominantly Black, publicly insured, and lived far from tertiary care centers [31]. Commentary on these findings emphasized that inadequate transportation and the multigenerational nature of poverty amplify survival gaps, underscoring that material hardship functions both as a biologic stressor and a logistical barrier [32]. Qualitative studies convey the lived experience of these structural inequalities. Residents of Walker County cited cost of care, lack of insurance, and perceived poor quality of local services as major barriers to prevention [27]. In colorectal cancer surveillance, patients with higher educational attainment were significantly more likely to complete post-procedure colonoscopies, suggesting that literacy and empowerment translate into adherence [30]. These data reveal a continuous gradient from economic context to health behavior to clinical outcome.

Financial scarcity limits screening, missed screening delays diagnosis, and delayed diagnosis worsens prognosis and increases treatment cost, perpetuating the cycle of disadvantage. Alabama’s economic geography reinforces these challenges. Rural counties dependent on low-wage industries and facing hospital closures shift travel and financial burdens onto patients. In these settings, poverty itself becomes an exposure that shapes both cancer risk and recovery. Within the Robison and Hudson framework, these dynamics illustrate the convergence of patient-level factors such as income, education, and coping resources with system-level factors including provider distribution, affordability, and continuity of care [24]. When patient vulnerability meets system insufficiency, morbidity compounds. Provider communication and follow-up behavior further modulate this relationship, making the patient-provider-system triad central to both prevention and survivorship outcomes.

### Racial disparities, community burden, and environmental risk

Factors associated with self-reported race and place emerge as interconnected determinants of cancer risk and survivorship across nearly all studies. Black Alabamians experience higher colon cancer incidence and are more likely to present with distant-stage disease, even after adjusting for county-level poverty [35]. These findings highlight the persistence of racialized exposure and systemic barriers. Among HIV-positive Black women, internal motivation and perceived susceptibility encouraged cervical cancer screening, but these positive drivers were outweighed by inadequate knowledge, transportation challenges, and low perceived risk, which acted as structural and cultural barriers to follow-through [43]. Racial disparities are also evident in pediatric oncology. Black children diagnosed with high-risk acute lymphoblastic leukemia had poorer survival outcomes than White peers, despite similar insurance coverage and poverty levels. This suggests the influence of biologic variation, differential treatment response, or provider bias [44]. Geographic and socioeconomic factors further compound these disparities. Counties marked by persistent poverty and long travel distances are home to a disproportionate number of Black children facing the highest mortality rates [31].

Community-based studies provide insight into how these disparities are experienced in everyday life. In Montgomery, Black adults, particularly women and those with lower education levels, expressed heightened concern about neighborhood pollution and its economic consequences [28]. In Mobile County, minority workers acknowledged elevated disease rates but avoided activism due to fears of jeopardizing employment in local industries [41]. Among Southeast Asian immigrants along the Gulf Coast, language barriers and cultural norms limited cancer screening uptake, even when risk factors were known [45, 46]. A multi-state review of cervical cancer prevention concluded that, despite national initiatives, medically underserved racial and ethnic groups continue to bear a disproportionate burden of disease [47]. These findings portray inequity as a layered exposure that is biological, social, and environmental. Minority communities face greater contact with pollutants, fewer economic options for relocation, and reduced access to culturally competent care.

Interpreted through the Robison and Hudson framework, racial disparities reflect a complex interplay of patient-level factors such as cultural norms, trust, and socioeconomic constraints; provider-level factors, including communication quality, implicit bias, and cultural competency; and system-level factors such as geographic maldistribution of services, insurance inequities, and regulatory enforcement [24]. Differences in tumor biology may further exacerbate these disparities. The interaction of limited patient agency, variable provider responsiveness, and systemic underinvestment contributes to the excess morbidity observed among racial and ethnic minorities in Alabama and stresses the need for integrated, equity-focused survivorship strategies that combine environmental justice with clinical reform.

## Discussion

This scoping review examined environmental, social, and structural determinants of cancer risk and outcomes in the state of Alabama. Across 21 studies published between 2002 and 2024, cancer in Alabama is shown to be deeply connected to geography and history. As summarized in Table 2, several environmental, social, and structural determinants were consistently reported across multiple cancer types, suggesting that cancer burden in Alabama is shaped by overlapping multilevel factors rather than isolated disease-specific processes. The state’s industrial legacy, persistent poverty, and racial segregation collectively shape who develops cancer, the stage at which it is diagnosed, and the likelihood of surviving beyond treatment. The current discussion is organized through the framework of the Robison and Hudson survivorship framework, which emphasizes multiple levels of influence; patient, provider, cancer burden, and healthcare system, on outcomes across the cancer continuum [24]. Using this framework allows us to examine how environmental exposures, social determinants, and structural inequities intersect to shape cancer outcomes in Alabama.

Cancer outcomes in Alabama reflect a complex interplay of environmental exposures, socioeconomic deprivation, and structural inequities. At the individual level, patient experiences, self-efficacy, access to care, the built environment, and physiological factors affect both cancer risk and survivorship. Environmental exposures such as air pollution and food insecurity interact with psychosocial and behavioral factors to influence disease onset and outcomes. Industrial emissions in Decatur, Jefferson, and Mobile Counties have been linked to elevated rates of bladder, liver, and ovarian cancers [33, 39, 40]. National evidence further supports these associations, demonstrating that ambient air pollution and persistent industrial chemicals are linked to increased risks of lung and hormonally mediated cancers [18, 20]. Persistent chemicals such as PFAS, phenols, and parabens further extend risk, with biomarkers associated with higher odds of melanoma, ovarian, and uterine cancers among women [21].

The state’s long industrial history, particularly in Jefferson County and North Birmingham, has produced decades of air, water, and soil contamination from steel and coke production facilities. Coke oven emissions, recognized as human carcinogens, remain a significant concern [48, 49]. Facilities such as the ABC Coke plant, US Pipe and Foundry, and Alabama Gas Company have been identified as major sources of local pollution, with more than 1.9 million pounds of chemical emissions reported in 2017, ranking Alabama 10th nationally in toxic releases per square mile [50, 51]. These exposures disproportionately affect predominantly African American neighborhoods already burdened by poverty and limited healthcare access [7, 52, 53].

Food and neighborhood environments also act as indirect carcinogenic exposures. Audits in 12 Black Belt cities revealed convenience stores dominate, offering calorie-dense foods with little access to fresh produce [26]. Residents of Walker County cited high food costs, limited insurance, and scarce primary care as barriers [27]. These findings illustrate how economic deprivation and poor infrastructure transform diet into an environmental exposure, supported by evidence linking “food swamps” to obesity-related cancer mortality [54] and chronic inflammation [55].

The framework also emphasizes the role of perceptions and self-efficacy in shaping health behaviors [24]. Gerbi et al. (2011) found African American women in Montgomery expressed concern about pollution but lacked resources to act, while lower education correlated with fatalistic views [28]. This aligns with literature documenting fatalism and perceived helplessness among marginalized groups facing chronic exposure [11, 56]. Fatalism reduces participation in screening, not from ignorance but systemic disempowerment [57]. Enhancing environmental health literacy through tailored education and faith-based outreach could strengthen empowerment, consistent with NIEHS recommendations [10, 47] and cancer survivorship frameworks [58].

Geography and access further compound these challenges. Rural residents travel farther for cancer treatment than urban patients, particularly those with prostate cancer [29]. Children in counties with persistent poverty and long distances from tertiary care centers experience higher five-year mortality [31, 32]. The absence of pediatricians in more than one-third of Alabama counties [59] adds to this risk. Black Alabamians disproportionately experience physical and social exposures, including proximity to industrial zones and limited access to nutritious foods [35, 47, 60]. Moberg’s ethnography in North Mobile County illustrates how economic dependence on industry suppresses activism [41]. These intersecting burdens form a cumulative exposure pathway across the life course.

The next element in the Robison and Hudson framework is provider knowledge, communication, and advocacy. Despite growing awareness of environmental carcinogenesis, integration of exposure histories into clinical care remains rare [61]. Few Alabama studies document clinicians incorporating environmental data or counseling, reflecting national gaps where risk is seldom included in electronic health records or survivorship tools [62]. Robison and Hudson emphasize that addressing lifelong risks requires clinicians to recognize environmental contributors to morbidity, a missed opportunity to connect prevention and survivorship [24]. Provider communication and trust are equally critical. In Walker County, residents reported frustration with dismissive communication about cancer and environmental risk [27]. Among HIV-positive African American women, poor provider communication and transportation barriers hindered cervical cancer screening despite high motivation [43]. These findings mirror national studies showing mistrust rooted in historical inequities and medical racism erodes engagement in prevention and survivorship programs [52, 63]. Improving cultural competence and empathy can strengthen patient-provider relationships and adherence to follow-up care [64, 65].

Providers also serve as educators and advocates. In Alabama’s underserved regions, faith-based nurses and lay navigators have increased screening rates [27, 47]. Expanding their role to include environmental and nutritional risk communication would extend survivorship care into prevention. National programs, including NIEHS partnerships, show that community-based provider training improves recognition of environmental risks [10, 66]. Within the Robison and Hudson framework, this aligns with education and resource dimensions, transforming providers into proactive agents of health equity [67]. Beyond clinical encounters, provider engagement in community-based participatory research (CBPR) can amplify community voices and address data gaps. Cheung et al. (2023) demonstrated that geospatial cancer mapping combined with community engagement identifies localized clusters that inform prevention strategies [34]. Providers thus act as bridges between community observations and formal science, a key component of survivorship responsibility intended by Robison and Hudson [24].

Moreover, the framework addresses the cancer level, which reflects biological and clinical expressions of social and environmental exposures, encompassing incidence, mortality, and treatment outcomes. In Alabama, these outcomes reveal the combined influence of chronic exposures, socioeconomic deprivation, and racialized inequities. Cancer incidence and mortality are highest in areas where industrial, chemical, and agricultural exposures converge. Counties with dense pulp and paper manufacturing report higher ovarian cancer rates [33]. Occupational cohorts from Decatur’s fluorochemical facilities show excess mortality from bladder and liver cancers [39, 40], consistent with PFAS carcinogenicity [13]. Leukemia risk has been linked to cumulative exposure to 1,3-butadiene [38], emphasizing the long latency of chemically induced malignancies [42]. These findings position Alabama within a national continuum of industrially mediated cancer risk [14, 48].

Racial disparities amplify cancer burden. Black residents experience higher colon and cervical cancer incidence and are more likely to present with late-stage disease [35, 47, 60, 68]. African American men in Alabama and Mississippi have poorer prostate cancer survival due to delayed diagnosis and lower treatment intensity [69], while children of color experience inferior leukemia outcomes even when insured [44]. These inequities reflect both biologic susceptibility and systemic barriers such as limited access, mistrust, and provider bias [6, 44, 70]. Tumor behavior and survival are not purely clinical; they are socially conditioned expressions of exposure and inequity [71]. Survivorship outcomes remain uneven. Survivors facing economic hardship report greater distress, lower quality of life, and reduced follow-up compliance [43, 72]. Desmond et al. (2017) found that southern cancer survivors trail national averages in survivorship care metrics such as physical activity counseling and mental health referral [73]. These deficits reinforce the need for cancer-specific survivorship plans that integrate social and environmental dimensions. Emerging research on precision survivorship (Hiatt & Breen, 2018) suggests integrating environmental and genomic data to tailor interventions for high-risk populations [74].

Finally, the framework addresses the health care system level [24], where environmental inequity in Alabama is sustained by historical industrialization, weak policy enforcement, and chronic underinvestment in healthcare infrastructure. Industrial corridors from Jefferson County to Mobile show how economic dependence on hazardous industries limits reform [41, 75]. Persistent emissions and decades of soil and water contamination demonstrate how policy failure translates into health disparity [76]. Similar to Louisiana’s “Cancer Alley,” Alabama’s industrial geography reflects national patterns where race and poverty predict proximity to pollutants [77–79].

Socioeconomic deprivation compounds these weak-nesses. Counties with fewer physicians, lower educational attainment, and higher poverty experience later-stage diagnoses and higher mortality [35]. Persistent poverty correlates with reduced political voice, limiting advocacy for cleanup or healthcare expansion [31, 32]. Hospital closures and long travel distances exacerbate treatment discontinuity [29, 80]. These structural deficits embody Robison and Hudson’s “capacity” construct, the system’s ability, or inability, to mitigate cumulative risk [24].

### Environmental risk as a survivorship care concern

Although environmental risks are frequently discussed in relation to cancer incidence and mortality, the findings of this review suggest that these exposures also have important implications for survivorship care and long-term outcomes. Increasing evidence indicates that environmental and place-based exposures influence not only cancer development but also treatment burden, comorbidity, recurrence risk, quality of life, and survivorship trajectories [81, 82]. Survivors residing in environmentally burdened or resource-limited communities may experience cumulative disadvantage through continued exposure to pollutants, reduced access to healthy environments, and barriers to ongoing care and health-promoting behaviors.

Environmental risks may shape survivorship through multiple pathways. Chronic exposure to air pollution and environmental contaminants has been associated with inflammation, cardiopulmonary disease, and other chronic conditions that may complicate cancer recovery and survivorship outcomes [83, 84]. These concerns are particularly relevant given the high burden of comorbidities observed across several studies included in this review. In addition, rural and socioeconomically disadvantaged communities often experience limited access to healthcare infrastructure, healthy food environments, physical activity resources, and specialty survivorship services, all of which influence long-term survivorship care and health outcomes [85, 86].

The overlap between environmental burden and structural disadvantage is especially important in Alabama, where persistent poverty, rurality, and healthcare workforce shortages intersect with environmental exposures and cancer disparities. Evidence from the Deep South suggests that communities experiencing high cancer burden also face transportation barriers, limited preventive care access, and constrained community resources [87]. These factors may impair adherence to follow-up care, surveillance, management of late and long-term effects, and chronic disease management after treatment completion. Environmental context may also influence survivorship through behavioral pathways. Food environments and neighborhood conditions shape dietary behaviors, physical activity, and other modifiable risk factors associated with recurrence and chronic disease burden [88]. Survivors living in underserved communities may therefore face ongoing exposure to conditions that increase vulnerability to poor survivorship outcomes even after treatment ends.

Collectively, these findings support a broader conceptualization of survivorship care in which environmental and structural conditions are recognized as part of the survivorship experience rather than external contextual factors. This interpretation aligns with quality cancer survivorship care frameworks that emphasize surveillance, management of long-term and late effects, health promotion, care coordination, and patient-centered survivorship outcomes [89]. In Alabama, where environmental risks frequently overlap with rurality, poverty, and healthcare access limitations, integrating environmental risk assessment into survivorship care may help identify survivors at greater risk for adverse outcomes and guide more contextually responsive follow-up care. These relationships are conceptually illustrated in Fig. 4, which adapts principles from the survivorship care framework proposed by Nekhlyudov and Colleagues (2019) to contextualize the influence of environmental, social, and structural determinants on cancer outcomes and survivorship in Alabama [89].

Across all levels, survivorship in Alabama reflects a cumulative burden shaped by environmental exposures, socioeconomic disadvantage, and structural inequities. These interconnected determinants influence cancer risk, treatment access and adherence, survivorship care, and long-term quality of life. Addressing these challenges will require multilevel strategies that strengthen environmental health literacy, improve integration of environmental exposure assessment into clinical and survivorship care, expand access to supportive services, and address structural barriers that contribute to disparities across the cancer continuum. Future research should prioritize survivorship-focused approaches that incorporate environmental, social, and clinical data to better understand how place-based exposures influence long-term cancer outcomes and survivorship experiences in high-burden populations.

### Implications for policy, practice, and research

Policy reform must bridge environmental regulation, healthcare infrastructure, and social justice. Integrating Alabama’s cancer registry with Toxic Release Inventory data could enable real-time identification of emerging clusters [90]. State mandates for cumulative risk assessment and environmental equity screening would align with NIEHS and EPA guidance [10, 91]. Investments in clean water, air-quality monitoring, and rural healthcare infrastructure, especially tele-oncology [92], would help reduce both exposure and access inequities. Financial barriers perpetuate inequity. Survivors and families face significant out-of-pocket costs for transportation, medication, and ongoing care, contributing to financial toxicity [5]. Policies ensuring comprehensive insurance coverage and financial navigation services can buffer against these burdens, reinforcing survivorship as both a medical and social responsibility. Structural racism remains a fundamental determinant. As Krieger and Bullard argue [56, 93], unequal power relations determine who bears environmental risks and who benefits from protection. Alabama’s predominantly Black communities near industrial sites face higher exposure and weaker regulatory enforcement, confirming that environmental justice is inseparable from cancer prevention and survivorship equity.

Clinicians should incorporate environmental and social screening into oncology and primary care workflows. Survivorship care plans need to address exposure reduction, nutrition, transportation, and mental health support, consistent with Deep South survivorship recommendations [73]. Community-based participatory research models, such as those utilized in Theresa and Colleagues (2011), empower residents to participate in data collection and advocacy [87]. Faith-based coalitions have also shown promise in sustaining outreach and education in underserved areas [27]. Expanding tele-oncology networks is critical in regions where pediatric and oncology specialists are scarce [92].

Future studies should integrate geospatial exposure data, biomarkers, and clinical outcomes to establish dose-response relationships for PFAS and other carcinogens. Longitudinal cohorts, such as the Southern Community Cohort Study, offer models for examining gene-environment interactions over time [94]. Implementation research should evaluate whether environmental policy interventions such as emission controls or improved food access reduce cancer incidence or mortality. Climate resilience metrics should also be incorporated, recognizing that flooding and heat can mobilize contaminants and disrupt care delivery [95].

### Broader relevance and transferability

Alabama’s environmental cancer burden mirrors regional inequities across the Gulf South, Appalachia, and the industrial Midwest. Coal ash exposure in Kentucky and West Virginia and heavy metal pollution in Michigan reveal similar pathways from legacy industry to health disparities [78, 96]. Alabama’s integration of geospatial mapping, community engagement, and justice framing offers a replicable model for other states. These insights align with the National Cancer Plan (2023) [97] and EPA EJScreen priorities that link cancer prevention with environmental and climate policy [91]. Health equity cannot be achieved without environmental equity.

### Limitations and future directions

The reviewed evidence varies in design and rigor; most studies are ecological, limiting causal inference. Exposure measurement for PFAS and butadiene is incomplete, and community-driven studies remain scarce. Future research should pursue longitudinal cohort linkage, participatory design, and integration of climate resilience metrics. Recognizing that environmental disruptions such as flooding and heat waves can mobilize contaminants and interrupt care is essential [98]. Cross-disciplinary collaboration between environmental scientists, oncologists, and policy experts is crucial for translating evidence into prevention.

## Conclusions

The evidence reviewed shows that cancer outcomes in Alabama are shaped by a complex interplay of environmental, social, and structural factors. Industrial contamination, food insecurity, healthcare scarcity, and racial inequities converge to produce a landscape of cumulative vulnerability. Within Robison and Hudson’s framework [24], patient behaviors, provider capacity, and system structure operate as inter-locking determinants of survivorship. Achieving equity, therefore, requires coordinated remediation, equitable infrastructure, culturally competent care, and robust survivorship planning. Place is not merely where health happens; it is a determinant of health itself. Advancing environmental justice and cancer prevention together is essential to narrowing Alabama’s cancer gap and achieving equitable outcomes across the Deep South.

## Figures and Tables

**Fig. 1:**
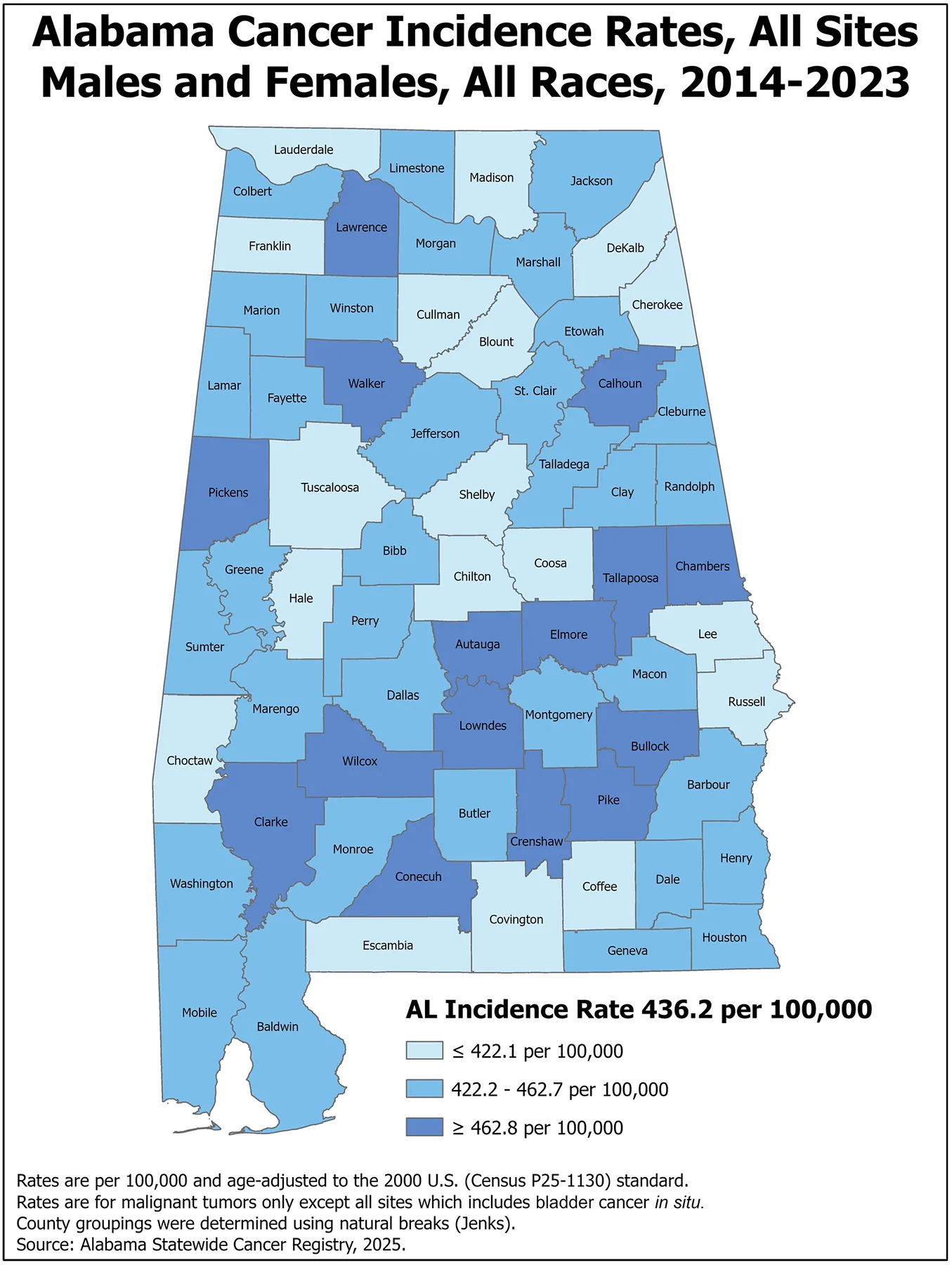
Alabama cancer map

**Fig. 2:**
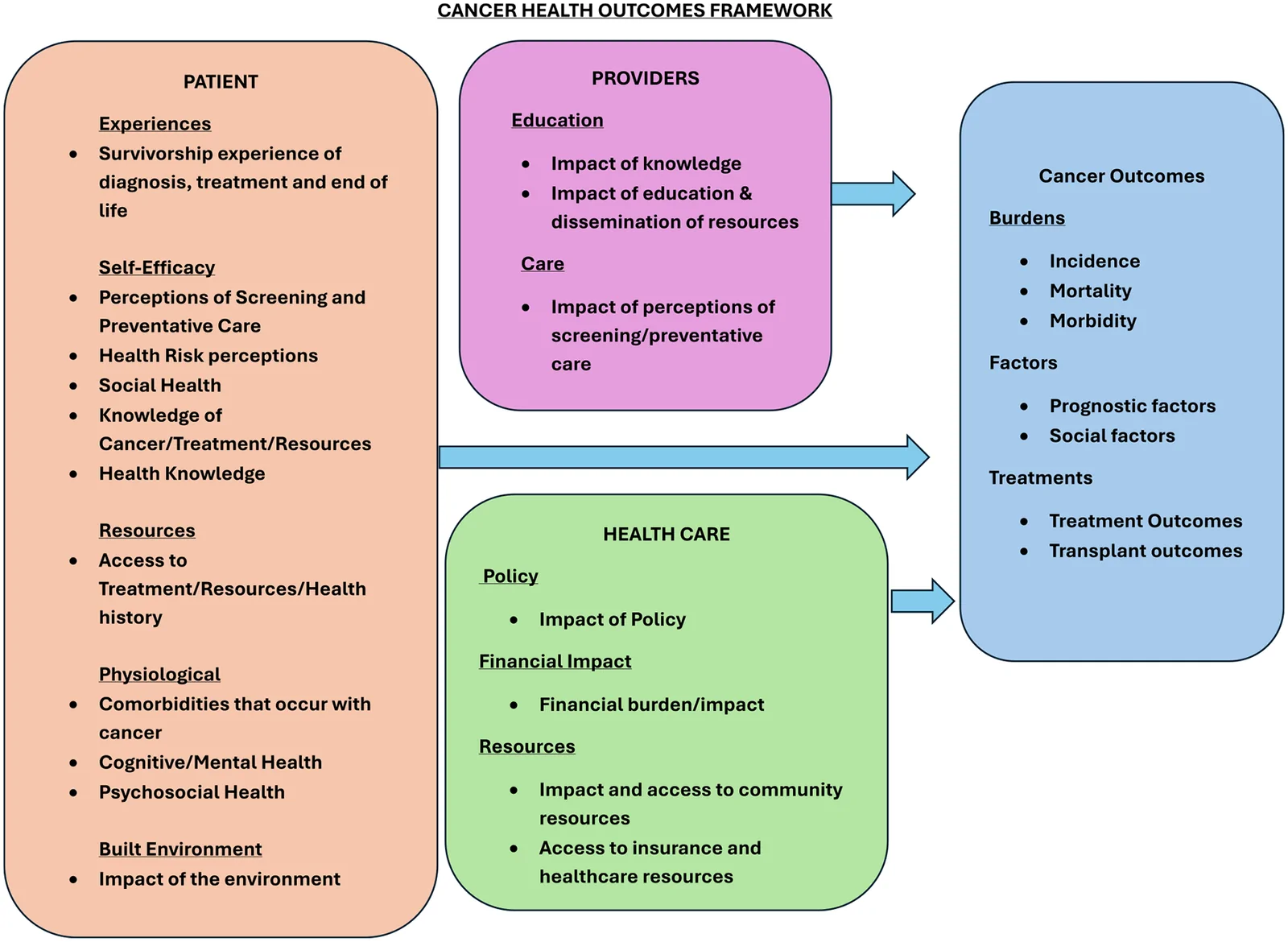
Framework modified from Robinson and Hudson (2014) [24]

**Fig. 3:**
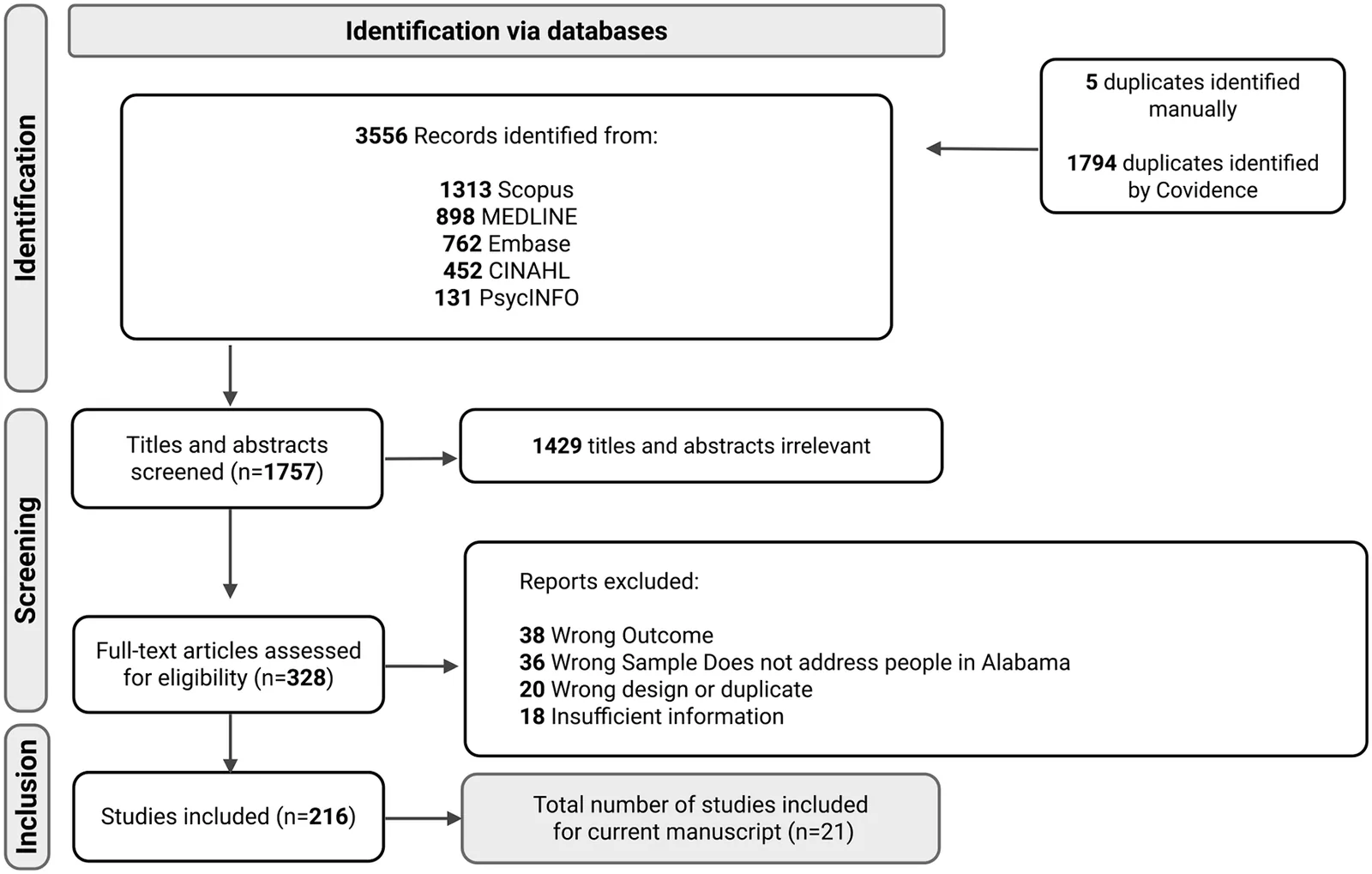
PRISMA flow diagram

**Fig. 4:**
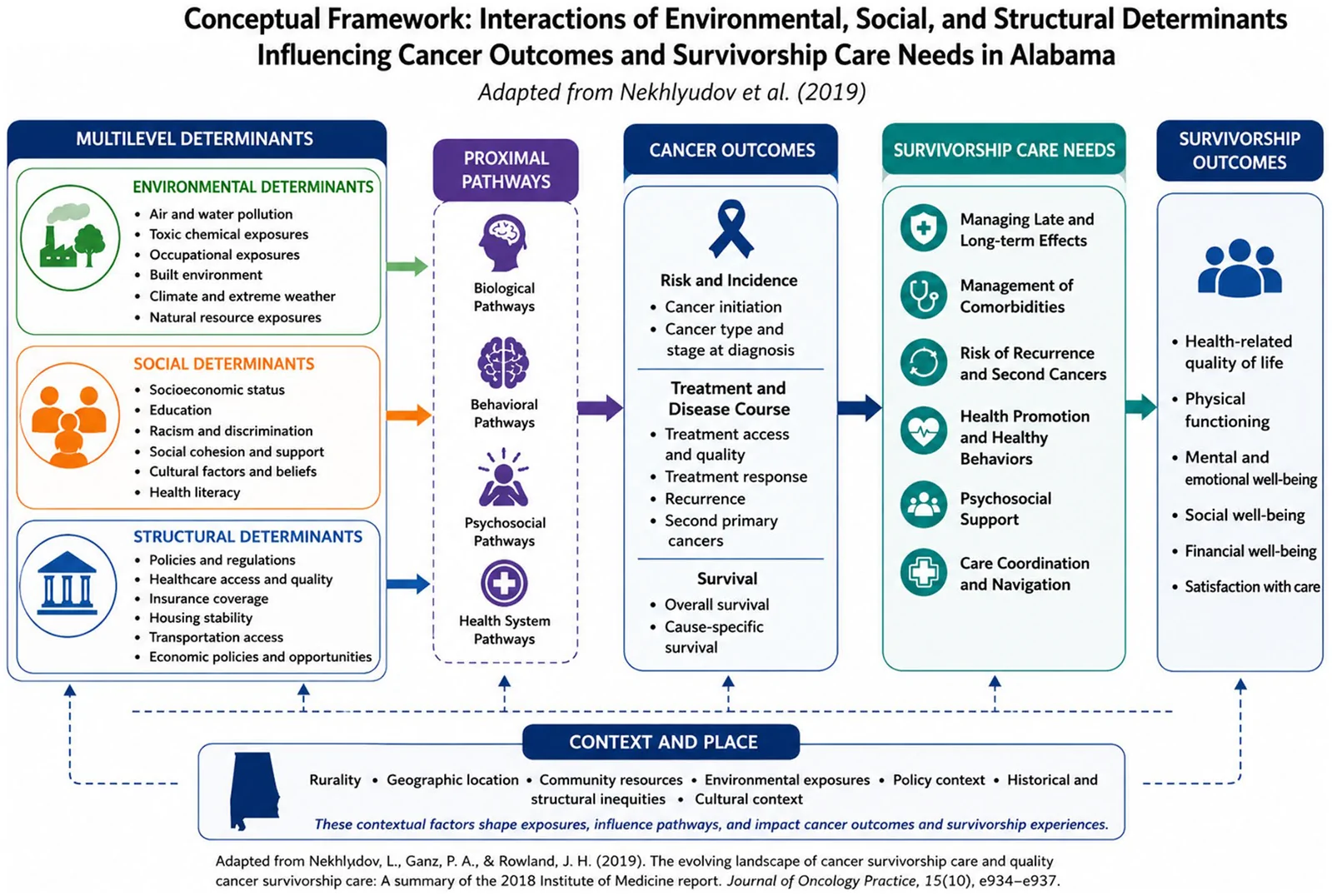
Conceptual framework: interactions of environmental, social, and structural determinants influencing cancer outcomes and survivorship care needs in Alabama. Adapted from Nekhlyudov et al. (2019)

**Table 1:** Characteristics of studies included in the systematic review of environmental risks on cancer outcomes in Alabama

Author (Year)	Cancer type	Population	Purpose	Sample/design	Key findings	Funding source	Theme(s)*
Moberg (2002)	General cancer/environmental health	North Mobile County, AL	Explore community attitudes toward industrial pollution	42 residents/workers; Qualitative	Residents accepted high cancer rates to preserve jobs, reflecting economic dependence and muted advocacy	Not reported	3, 6
Alexander et al. (2003)	Bladder	Decatur, AL fluorochemical workers	Evaluate mortality among long-term chemical workers	2083 workers; Retrospective cohort	High-exposure jobs associated with increased bladder-cancer deaths	Industry-funded	2, 3, 4
Olsen et al. (2004)	Liver/biliary tract (plus other outcomes)	Decatur, AL fluorochemical workers	Compare chemical vs film plant workers	1311 workers across groups; Cohort	Elevated liver/biliary disorders and some cancers in chemical unit employees	Industry-funded	2, 3
Shipp et al. (2005)	Colon/Colorectal	Alabama residents	SES, race, physician density vs CRC incidence/stage	5,788 cases (1996–1999); Secondary analysis	Higher incidence and distant disease tied to poverty, lower physician density, and Black race	Not reported	2, 5, 6
Xu et al. (2005a)	General cancer risk	Vietnamese Americans in rural AL	Identify aggregate-specific cancer risk factors	284 adults; Quantitative	Elevated behavioral/environmental risks vs national data	Not reported	6
Xu et al. (2005b)	General cancer risk	Southeast Asian Americans, Gulf Coast	Profile cancer risk factors, prevention barriers	332 adults; Quantitative	Cultural/linguistic barriers highlight need for tailored interventions	Univ./Business grant	6
Joseph et al. (2007)	General cancer	AL and Gulf Coast states	Estimate burden post-Hurricane Katrina	Registry data; Secondary analysis	Overall, AL incidence lower than USA, but displacement complicated care/registry continuity	Not reported	2
Scarinci et al. (2010)	Cervical	Multistate underserved populations incl. AL	Review prevention delivery in high-burden groups	Review/mixed	Persistent barriers (cost, mistrust, access) keep burden high; outreach helps	NIH/NCI	6
Gerbi et al. (2011)	General cancer	African Americans, Montgomery, AL	Psychosocial responses to perceived environmental risk	145 adults; Quantitative	Women and lower-education participants more concerned but with limited efficacy	Not reported	1, 6
Gyawu et al. (2015)	General cancer/prevention	Alabama Black Belt	Food environment audit and cancer-prevention opportunity	12 cities in 5 counties; Field audit	Scarce healthy foods and minimal health messaging; obesogenic retail mix	Private/Org	1
Graves et al. (2015)	General health/cancer risk	Walker County, AL	CBPR to identify health priorities	774 residents; Mixed-methods	Poor access, cost, obesity, and weak primary care seen as cancer-related challenges	Community/Org	1, 5
Williams et al. (2015)	Cervical	HIV-infected African American women, AL	Sociocultural/structural factors in screening	20 women; Qualitative	Knowledge gaps, transport, and perceived susceptibility shaped uptake	NIH/NCI	6
Sielken and Valdez-Flores (2015)	Leukemia/lymphoid neoplasms	SBR industry workers (incl. AL)	Model 1,3-butadiene exposure-response	Occupational cohort; Secondary	Clear dose-response between cumulative BD and leukemia risk; ambient implications	Not reported	2, 3, 4
Walsh et al. (2017)	Leukemia (ALL, pediatric)	Alabama children	Racial/SES influences on outcomes	222 patients; Retrospective	Black children had poorer survival not explained by SES alone	Not reported	6
Wills et al. (2017)	Breast/Prostate/Colorectal	Deep South older adults (incl. AL)	Travel distance to treatment	36,786 Medicare claims; Quantitative	Rural Deep South patients traveled farther; longest for prostate cancer	Not reported	1
Hanchette et al. (2018)	Ovarian	Southeastern US counties (incl. AL)	Map ovarian cancer vs TRI paper-mill emissions	County-level spatial analysis	High ovarian-cancer clusters co-located with pulp/paper emissions	Foundation	2, 3, 4
Orloff et al. (2020)	Melanoma (uveal)	AL, NY, NC	Identify unusual regional accumulations	25 cases; Spatial clustering	Localized clusters warrant environmental investigation	State grant	2
Cheung et al. (2023)	Multiple myeloma	USA (incl. AL)	Geospatial hot-spot mapping	County-level modeling	Significant Southeast clusters suggest environmental and racial disparity patterns	NIH/NCI	2, 4
Hoppmann et al. (2023)	Pediatric cancers	Alabama children	Persistent child poverty and survival	2089 patients; Registry cohort	Persistent poverty linked to 30% higher 5-yr mortality; associated with race, distance	NIH/NCI & Foundation	1, 5, 6
Orjuela-Grimm and Beauchemin (2023)	Pediatric cancer	Alabama children	Contextualize poverty-mortality links	Commentary	Highlights transport/infrastructure and intergenerational poverty as survival barriers	Not reported	1, 5
Vashi et al. (2024)	Colon/Colorectal	AL patients post-EMR	Predictors of surveillance colonoscopy adherence	465 patients; Retrospective	Adherence varied by distance and education; moderate distances showed better return	Not reported	1, 5

* Theme legend: 1, food and neighborhood environment; 2, exposure assessment and environmental measurement methods; 3, industrial and toxic releases as exposures; 4, legacy and historical exposures and hematologic outcomes; 5, socioeconomic environment and cancer incidence; 6, racial disparities, community burden, and environmental risk

**Table 2:** Common environmental, social, and structural factors reported across major cancer types in Alabama

Cancer type	Environmental/behavioral factors reported	Social/structural factors reported	Cancer burden outcomes highlighted
Breast cancer [29]	Treatment access and travel-related barriers	Rural residence, distance to specialty care, healthcare access limitations among older adults in the Deep South	Treatment access burden and survivorship-related care challenges
Prostate cancer [4, 29]	Treatment access and travel burden	Rural residence, specialty care access limitations, geographic barriers to care, information-seeking barriers among African American men	Increased travel burden and disparities in care access
Colorectal/ Colon cancer [29, 30, 35]	Diet-related risk context, food environment, obesogenic retail environments, surveillance adherence barriers	Poverty, lower physician density, Black race, education, distance to care	Higher incidence, distant-stage disease, and variation in surveillance colonoscopy adherence
Cervical cancer [43, 47]	Screening and prevention barriers	Cost, mistrust, transportation challenges, knowledge gaps, limited preventive care access among underserved populations	Persistent screening barriers and continued burden among underserved populations
Bladder cancer [39]	Occupational fluorochemical exposure	Industrial employment and workplace exposure	Increased bladder cancer mortality among high-exposure workers
Liver and biliary tract cancers [40]	Chemical plant exposure	Occupational exposure in industrial work settings	Elevated liver and biliary disorders and cancer-related outcomes among exposed workers
Leukemia/lymphoid neoplasms [38, 44]	1,3-butadiene exposure and industrial toxic exposures	Occupational and historical industrial exposure contexts, racial disparities	Dose-response relationship between cumulative exposure and leukemia risk; poorer survival among Black children
Pediatric cancers [31, 32, 44]	Community-level disadvantage and barriers to care access	Persistent child poverty, race, transportation barriers, infrastructure limitations	Higher 5-year mortality among children living in persistent-poverty communities
Ovarian cancer [33]	Toxic release inventory emissions from pulp and paper facilities	County-level industrial exposure patterns	Geographic clustering of ovarian cancer near industrial emission sites
Multiple myeloma [34]	Geographic environmental exposure patterns	Regional and racial disparity patterns across Southeastern counties	Significant geographic clustering suggesting environmental and racial disparity patterns
Melanoma/uveal melanoma [37]	Possible localized environmental exposure concerns	Regional clustering patterns warranting further investigation	Unusual geographic accumulation of cases
General cancer/cancer risk /Environmental cancer burden [26–28, 36, 41, 45, 46]	Industrial pollution, obesogenic food environments, disaster-related disruption, perceived environmental risk	Economic dependence on industry, cultural and linguistic barriers, low health literacy, weak primary care access, community mistrust, displacement-related care disruption	Elevated perceived cancer risk, prevention barriers, disrupted care continuity, and limited community advocacy

## Data Availability

No datasets were generated or analysed during the current study.
